# High-Fat Diet Changes Hippocampal Apolipoprotein E (ApoE) in a Genotype- and Carbohydrate-Dependent Manner in Mice

**DOI:** 10.1371/journal.pone.0148099

**Published:** 2016-02-01

**Authors:** Courtney Lane-Donovan, Joachim Herz

**Affiliations:** 1 Department of Molecular Genetics, University of Texas Southwestern Medical Center, Dallas, Texas, 75390, United States of America; 2 Department of Neuroscience, University of Texas Southwestern Medical Center, Dallas, Texas, 75390, United States of America; 3 Center for Translational Neurodegeneration Research, University of Texas Southwestern Medical Center, Dallas, Texas, 75390, United States of America; 4 Department of Neurology and Neurotherapeutics, University of Texas Southwestern Medical Center, Dallas, Texas, 75390, United States of America; 5 Center for Neuroscience, Department of Neuroanatomy, Albert-Ludwigs-University, Freiburg, Germany; Monash University, AUSTRALIA

## Abstract

Alzheimer’s disease is a currently incurable neurodegenerative disease affecting millions of individuals worldwide. Risk factors for Alzheimer’s disease include genetic risk factors, such as possession of ε4 allele of apolipoprotein E (ApoE4) over the risk-neutral ApoE3 allele, and lifestyle risk factors, such as diet and exercise. The intersection of these two sources of disease risk is not well understood. We investigated the impact of diet on ApoE levels by feeding wildtype, ApoE3, and ApoE4 targeted replacement (TR) mice with chow, high-fat, or ketogenic (high-fat, very-low-carbohydrate) diets. We found that high-fat diet affected both plasma and hippocampal levels of ApoE in an isoform-dependent manner, with high-fat diet causing a surprising reduction of hippocampal ApoE levels in ApoE3 TR mice. Conversely, the ketogenic diet had no effect on hippocampal ApoE. Our findings suggest that the use of dietary interventions to slow the progression AD should take ApoE genotype into consideration.

## Introduction

Alzheimer’s disease (AD) is the most prevalent dementia in individuals over 60 years of age, affecting around 30 million people worldwide. The most common genetic risk factor for AD is possession of the ε4 allele of apolipoprotein E (ApoE4) [1]. In addition to genetic risk factors like ApoE4, non-genetic risk factors have also been established for AD, including the lifestyle factors of weight, diet, and exercise, and these lifestyle factors may account for up to one-third of AD cases [2]. With the concurrence of both an aging population and a progressively overweight and obese society, understanding the intersection of diet, genotype, and cognitive decline will become increasingly important.

Several studies have probed the effect of modifying lifestyle factors to improve AD outcomes and their interaction with ApoE genotype. For example, engaging in daily exercise improves cognitive function in patients with AD and related cognitive impairment and reduces brain amyloid [3], and ApoE4 carriers experience greater positive benefits of exercise engagement [4]. Similar to exercise, the introduction of a Western diet and associated obesity to a country correlates with an increased incidence of AD in that country [5], while adherence to a Mediterranean diet is associated with a reduced risk of AD and mild cognitive impairment (MCI) [6]. However, in contrast to exercise, dietary interventions appear to be less effective in ApoE4 carriers [7].

In the plasma, ApoE plays a key role trafficking lipid-filled lipoproteins from the intestine to the liver, thus a high-fat diet increases plasma levels of ApoE [8]. However, the effect of diet on hippocampal levels of ApoE is not well understood. Here, we investigated the effects of dietary changes on hippocampal ApoE. We found that in stark contrast to plasma ApoE, high-fat diet either reduces or has no effect on hippocampal levels of ApoE, in an isoform-dependent manner. Moreover, this effect is carbohydrate-dependent, as a high-fat, no-carbohydrate, ketogenic diet did not affect hippocampal levels of ApoE. Overall, these findings suggest that diet can have a direct effect on hippocampal ApoE and that dietary interventions for AD should take into consideration ApoE genotype.

## Materials and Methods

### Animals and Ethics Statement

All animal care protocols were followed in accordance with and approved by the Institutional Animal Care and Use Committee of the University of Texas Southwestern Medical Center. Mice were deeply anesthetized by isoflurane and then sacrificed. Wildtype (hybrid SV129/C57BL6) mice and ApoE3 [9] and ApoE4 [10] targeted replacement mice bred to homozygosity were used in this study. Animals were group-housed in a standard 12-h light cycle and fed ad libitum standard mouse chow.

### Ketogenic and High-fat diets

After weaning, animals were maintained on a chow (18% protein, Harlan Teklad #2018) diet. 2–3 months-old age-matched animals were fed either a high-fat (kcals: 60% fat, 20% protein, 20% carbohydrate, lard-based, D12492 from Research Diets, Inc.), ketogenic (75% fat, 8.6% protein, 3.2% carbohydrate, lard- and butter-based, AIN-76A-Modified from Bio Serv), or chow diet. Mice were fed ad libitum on all diets.

### Tissue collection, homogenization, and western blotting

Mice were anesthetized and blood was collected retro-orbitally using heparinized hematocrit tubes (Drummond). Samples were spun for 10 minutes at 4,000 rpm at 4C, and the supernatant (plasma) was retained. Mice were then deeply anesthetized by isoflurane and sacrificed, then transcardially perfused with ice-cold PBS. The hippocampi were dissected out and flash frozen in liquid nitrogen. Samples were diluted 1:20 or homogenized in RIPA buffer with protease and phosphatase inhibitors (Sigma). Proteins were resolved via polyacrylamide gel (Bio-Rad) and transferred to nitrocellulose membranes. Membranes were blocked in blocking buffer (Li-Cor), then incubated with primary antibody. Membranes were then treated with secondary antibody and proteins were visualized using Odyssey CLx (Li-Cor).

The following primary antibodies were used: goat polyclonal anti-ApoE (Calbiochem 1:1000), rabbit polyclonal anti-ApoE (199, generated previously by our lab, see [11] 1:1000), rabbit polyclonal anti-receptor-associated protein (RAP) (692, generated previously by our lab, see [12] 1:1000), and rabbit polyclonal anti-Transferrin (Abcam ab1223 1:1000). Secondary antibodies used were anti-rabbit 680 and anti-goat 800 (Li-Cor 1:3000).

### Statistical Analysis

Data were analyzed with GraphPad Prism software (version 6.0, GraphPad Software). Wild type mice were analyzed separately since a different antibody was used. WT samples were normalized to WT chow, and ApoE3/E3 and ApoE4/4 samples were normalized to ApoE3/E3 chow. Wildtype tissue samples were analyzed with unpaired two-tailed t-test. ApoE3 and ApoE4 tissue samples were analyzed using a two-way ANOVA. Similarly, body weight changes for all genotypes and diets were analyzed using a two-way ANOVA. For two-way ANOVAs, post-hoc analysis was performed using Bonferroni correction for multiple comparisons. Data are represented as mean ± S.E.M.

## Results

To test the effect of diet on ApoE levels, age-matched wildtype (WT), ApoE3/E3 targeted replacement (TR), and ApoE4/E4 TR male mice were fed a high-fat diet or chow diet for five-weeks. As expected, the high-fat diet induced weight gain in all genotypes. In this cohort, there was an overall effect of genotype on body weight; however, the post-hoc analysis did not reveal significant changes for any specific genotype-to-genotype comparison. It has previously been shown that ApoE4 TR mice have reduced weight compared to ApoE3 TR mice on both chow and high-fat diets [13]. This effect appears to increase with age, which may be why only a small difference is observed in our study. (Table 1.)

**Table 1:** High-fat diet (HFD) induces weight gain in wildtype, ApoE3, and ApoE4 mice.

Genotype	Wildtype	ApoE3/E3	ApoE4/E4
**Chow Diet (g)**	36.1 ± 1.6	33.4 ± 1.2	30.7 ± 0.6
**High-fat Diet (g)**	50.0 ± 1.9	47.6 ± 1.1	45.7 ± 1.7

A two-way ANOVA showed a significant effect of genotype (p = 0.0081), a highly significant effect of diet (p < 0.0001), and no interaction between genotype and diet (p = 0.9383). Post-hoc analysis revealed that all genotypes significantly gained weight on the high-fat diet (for WT, ApoE3, and ApoE4: p < 0.0001). Post-hoc analysis did not find a significant difference between genotypes within the chow or high-fat diet fed groups. WT:Chow (n = 4), WT:HFD (n = 4), ApoE3:Chow (n = 4), ApoE3:HFD (n = 5), ApoE4:Chow (n = 5), ApoE4:HFD (n = 4). The data are shown as mean body weight ± S.E.M.

**Fig 1:**
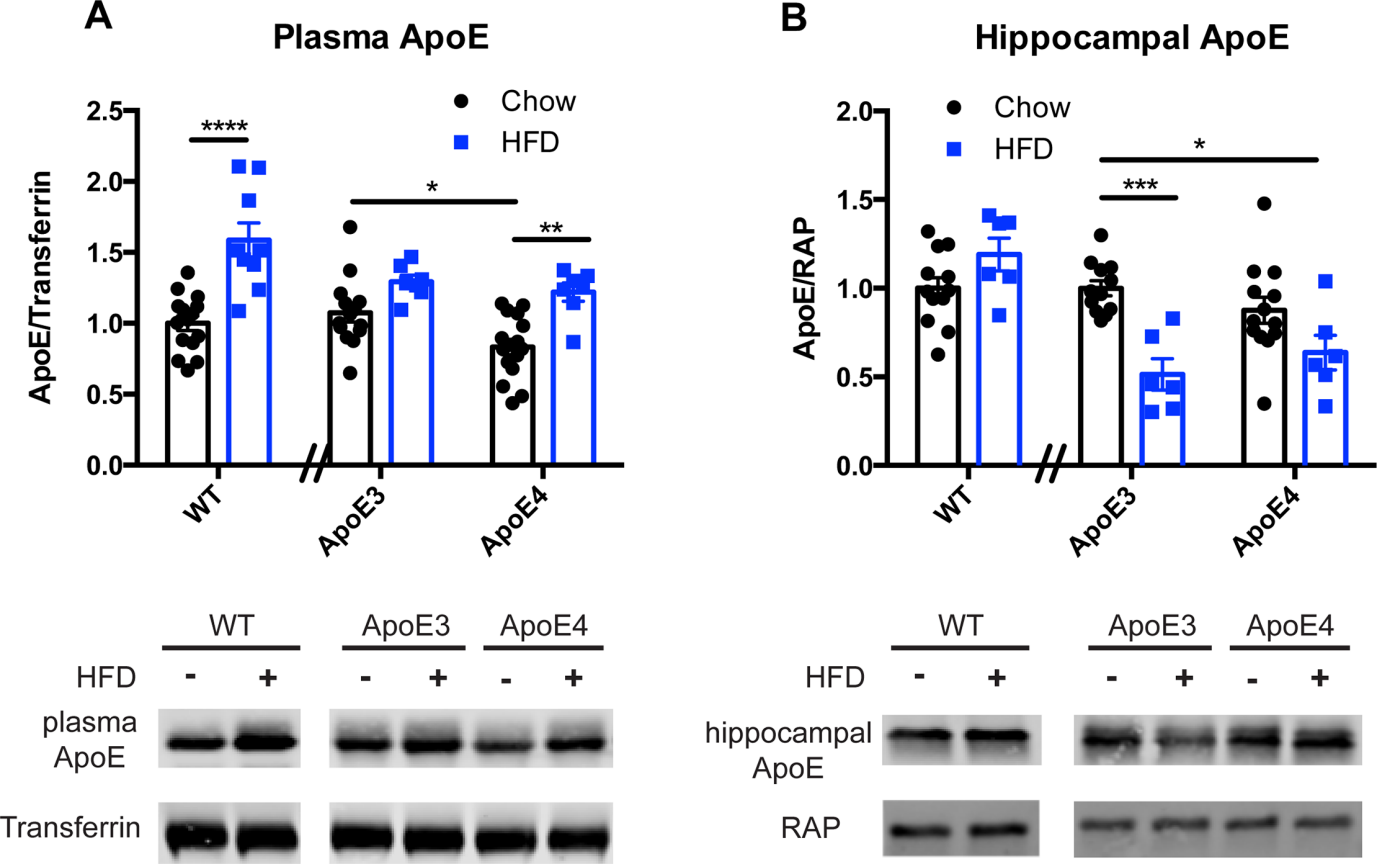
Effect of high-fat diet on plasma and hippocampal ApoE. **A.)** Plasma ApoE levels after 5 weeks on a high-fat or chow diet in wildtype, ApoE3 TR, and ApoE4 TR mice. WT:chow (n = 15), WT:HFD (n = 9), ApoE3:chow (n = 14), ApoE3:HFD (n = 7), ApoE4:chow (n = 16), ApoE4:HFD (n = 7). **B.)** Hippocampal ApoE levels after 5 weeks on a high-fat or chow diet in wildtype, ApoE3 TR, and ApoE4 TR mice. For all genotypes on HFD, n = 6. WT:chow (n = 12), ApoE3:chow (n = 12), ApoE4:chow (n = 13). Data are shown as mean ± S.E.M. (* = p < 0.05, ** = p < 0.01, *** = p < 0.001, **** = p < 0.0001).

Tissues from non-fasted mice were examined to determine the steady-state changes in ApoE caused by diet. Similar to prior studies [8,9,14], an increase in plasma ApoE was observed in response to diet. **Fig 1A.** For the wildtype mice, an unpaired t-test revealed a significant effect of diet on plasma ApoE (p < 0.0001). For the ApoE3 and ApoE4 mice, a two-way ANOVA revealed a significant effect of genotype (p = 0.0257), a significant effect of diet (p < 0.0001), and no interaction between diet and genotype (p = 0.2170). Post-hoc analysis revealed a significant effect of diet on plasma ApoE in ApoE4 (p < 0.01) mice. Moreover, post-hoc analysis revealed a difference in baseline plasma ApoE levels between chow-fed ApoE3 and ApoE4 mice (p < 0.05).

We then investigated diet-induced changes in the hippocampus, a region of the brain that is required for learning and memory and is affected early in AD [15]. In stark contrast to our plasma findings, high-fat diet reduced ApoE levels by approximately 50% in the hippocampus of ApoE3 TR mice, but caused no change in the ApoE4 TR mice. Diet also had no effect on hippocampal ApoE levels in WT mice. **Fig 1B.** For the wildtype mice, an unpaired t-test revealed no effect of diet on hippocampal ApoE (p = 0.0937). For the ApoE3 and ApoE4 mice, a two-way ANOVA revealed no effect of genotype (p = 0.9915), a significant effect of diet (p < 0.0001). Post-hoc analysis revealed a significant effect of diet on hippocampal ApoE in ApoE3 (p < 0.001) and no effect in ApoE4 (p > 0.05) mice. Additionally, post-hoc analysis revealed no difference in baseline hippocampal ApoE levels between chow-fed ApoE3 and ApoE4 mice (p > 0.05). No effect of diet or genotype was observed on receptor-associated protein (RAP), which was used as a loading control (two-way ANOVA, p_Genotype_ = 0.7547, p_Diet_ = 0.9376).

Several studies have suggested that the damaging effects of the high-fat diet come from the presence of carbohydrates in the diet, thus we examined the effect of ketogenic (high-fat, no-carbohydrate) diet on ApoE levels. In contrast to the high-fat diet, the ketogenic diet caused weight loss in the mice, and the mice equilibrated at a final weight that was 65–75% of the weight of chow-fed mice, which is similar to the weight loss observed in previous ketogenic studies [16]. (Table 2.)

**Table 2:** Ketogenic diet induces weight loss in wildtype, ApoE3, and ApoE4 mice.

Genotype	Wildtype	ApoE3/E3	ApoE4/E4
**Chow Diet (g)**	35.9 ± 3.0	38.34 ± 1.0	36.7 ± 1.1
**Ketogenic Diet (g)**	27.6 ± 1.0	24.7 ± 1.4	26.1 ± 0.7

WT:Chow (n = 7), WT:HFD (n = 8), ApoE3:Chow (n = 9), ApoE3:HFD (n = 6), ApoE4:Chow (n = 7), ApoE4:HFD (n = 7). A two-way ANOVA showed no effect of genotype (p = 0.968), a highly significant effect of diet (p < 0.0001), and no interaction between genotype and diet (p = 0.2225). A post-hoc analysis revealed a significant effect of diet on body weight each genotype [WT (p < 0.01), ApoE3 (p < 0.0001), ApoE4 (p < 0.001)]. Post-hoc analysis did not find a significant difference between genotypes within the chow or ketogenic diet-fed groups. The data are shown as mean body weight ± S.E.M.

**Fig 2:**
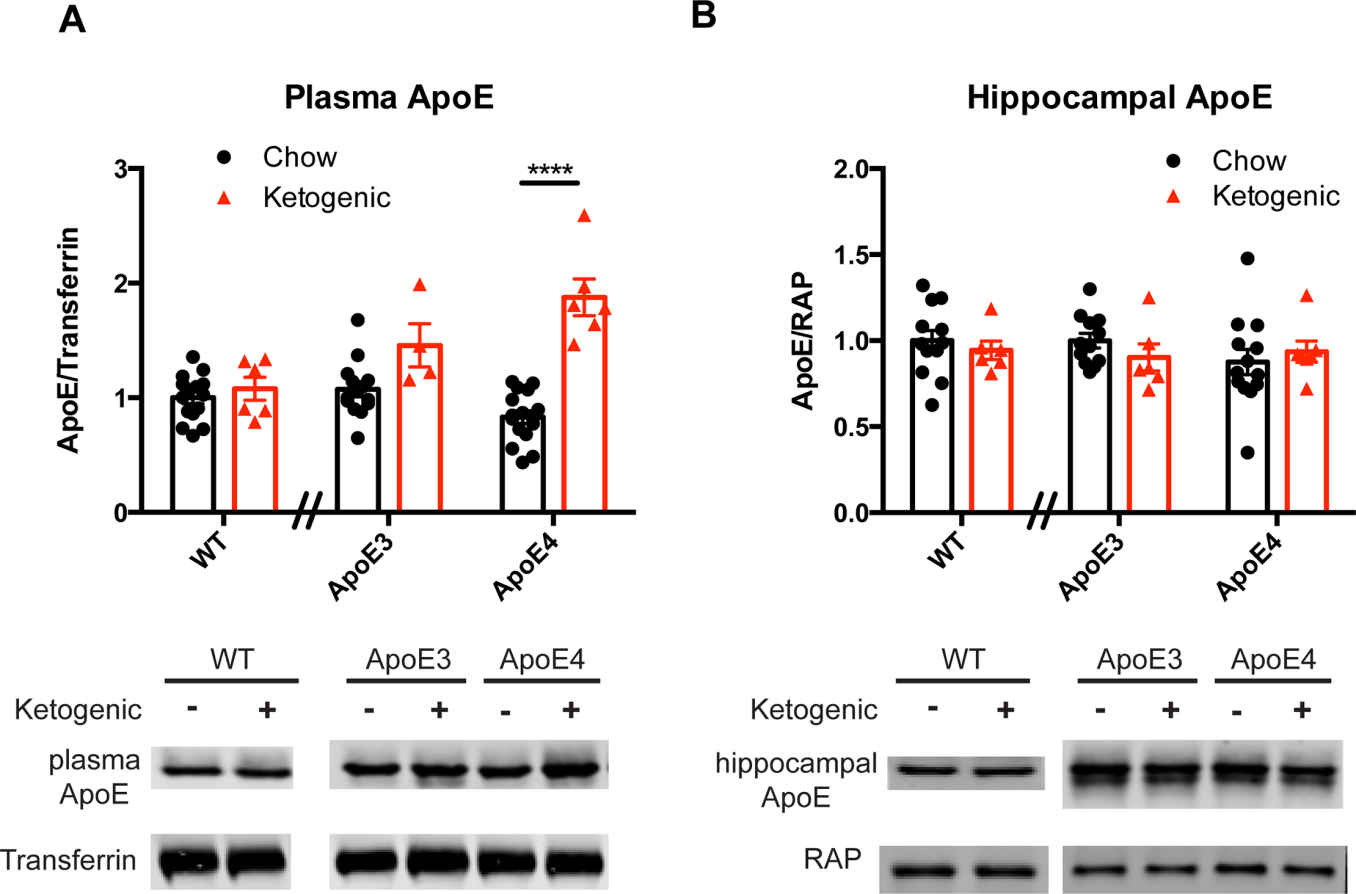
Effect of ketogenic diet on plasma and hippocampal ApoE. **A.)** Plasma ApoE levels after a 5 week ketogenic or chow diet in wildtype. WT:chow (n = 15), WT:keto (n = 6), ApoE3:chow (n = 14), ApoE3: keto (n = 4), ApoE4:chow (n = 16), ApoE4/E4 keto (n = 6). **B.)** Hippocampal ApoE levels after 5 weeks on ketogenic or chow diet. WT:chow (n = 6), WT:keto (n = 12), ApoE3:chow (n = 6), ApoE3: keto (n = 6), ApoE4: chow (n = 13), ApoE4: keto (n = 7). (**** = p < 0.0001). The data are shown as mean ± S.E.M.

The ketogenic diet had no effect on plasma ApoE levels in the wild type mice, but did cause a significant increase in the ApoE4/E4 mice that was higher than that induced by the high-fat diet. **Fig 2A**. For the wildtype mice, an unpaired t-test revealed no effect of diet on plasma ApoE (p = 0.8256). For the ApoE3 and ApoE4 mice, a two-way ANOVA revealed no effect of genotype (p = 0.3886), a significant effect of diet (p < 0.0001), and a significant interaction between diet and genotype (p = 0.0024). Post-hoc analysis revealed a significant effect of diet on plasma ApoE in ApoE4 mice (p < 0.0001) and no effect in ApoE3 mice (p > 0.05). Moreover, post-hoc analysis revealed no difference in baseline plasma ApoE levels between chow-fed ApoE3 and ApoE4 mice (p > 0.05).

Conversely, no effect of ketogenic diet on hippocampal ApoE was observed in any mouse line, suggesting that the effect of the high-fat diet on hippocampal ApoE is carbohydrate-dependent. **Fig 2B.** For the wildtype mice, an unpaired t-test revealed no effect of diet on hippocampal ApoE (p = 0.5622). For the ApoE3 and ApoE4 mice, a two-way ANOVA revealed no effect of genotype (p = 0.5196), no effect of diet (p = 0.7965), and no interaction between diet and genotype (p = 0.2645).

## Discussion

In this study, we investigated the intersecting effect of diet and genotype on ApoE expression in the brain. We found that a high-fat diet reduced hippocampal ApoE levels in mice expressing human ApoE3, but not ApoE4 or murine ApoE, and this effect was not observed after a similar amount of time on a ketogenic diet. Moreover, while a high-fat diet increased steady-state plasma ApoE in all genotypes, a ketogenic diet only increased plasma ApoE in human ApoE4 TR mice. Together, these results suggest that dietary composition alters ApoE protein levels in an isoform-specific manner.

Modulation of hippocampal ApoE levels by diet is not well understood. Multiple studies have shown that high-fat diet increases hippocampal ApoE mRNA expression in wild-type mice [17,18]; however, hippocampal levels of murine ApoE protein appear relatively unchanged by high-fat diet [18], a finding replicated here. Surprisingly, in contrast to the wildtype mice, we found that a high-fat diet significantly reduced levels of hippocampal human ApoE in ApoE3 TR mice, but not ApoE4 TR mice.

In peripheral lipid metabolism and trafficking, the key difference between ApoE4 and ApoE3 is the reduced ability of ApoE4 to participate in high-density lipoprotein (HDL) recycling, which results in impaired cholesterol efflux from cells and a reduced lipidation of ApoE4[19]. It is not currently technically possible to confirm that this ApoE4 effect occurs *in vivo* in the brain; however, it is likely that a similar process occurs in the CNS, with high-fat diet as an equalizer that occludes the isoform-specific differences on a low-fat diet. It has been shown that ApoE4 levels are reduced in the brain relative to ApoE3 at baseline[20]. In our experiments, we found a similar trend towards reduction of hippocampal ApoE in ApoE4 TR mice, which did not achieve significance. That said, it is tempting propose a model by which a high-fat diet brings levels of ApoE3 down to ApoE4 baseline levels.

To determine if the effect of the high-fat diet on hippocampal ApoE was purely due to dietary fat, we completed a comparable experiment in mice fed a ketogenic diet. The ketogenic diet, which is high-fat and very-low to no carbohydrate, causes increased energy expenditure and fatty acid oxidation with reduction in lipid synthesis [16]. The role of ApoE and its isoforms in the ketogenic state is relatively unknown. In contrast to the high-fat diet, we found no change in hippocampal ApoE levels in response to ketogenic diet for any genotype. However, we did observe an interesting effect of ketogenic diet on plasma ApoE, with ApoE4 mice having a large increase in plasma ApoE in response to ketosis, while ApoE3 mice experienced a more moderate change.

In rodents and humans, high-fat, western diets cause cognitive impairment [21,22]. The role ApoE genotype plays is relatively unknown, given that most studies of diet and Alzheimer’s are underpowered for such an analysis. Two interpretations are possible from our findings: either the high-fat diet-induced reduction in ApoE is itself harmful for brain function, or it is a protective adaptive response. In transgenic amyloid-β (Aβ)-overproducing mice, a model of AD, a high-fat diet increases Aβ accumulation and cognitive deficits. This can be reversed by treatment with a liver X receptor (LXR) agonist, which increases ApoE levels and stability [18]. On the other hand, genetic reduction of ApoE accelerates clearance of Aβ, and ApoE-reducing therapeutics are being explored for AD treatment [23,24]. To firmly establish the effect of diet by ApoE genotype, future studies will have to explore behavioral effects of high-fat diet on ApoE TR mice.

Our findings that a high-fat diet reduces hippocampal ApoE in mice in an isoform-dependent manner suggest that the effectiveness of dietary changes in AD patients may be affected by ApoE genotype. Our results encourage future studies on the effect of other dietary interventions, such as the Mediterranean diet, on hippocampal ApoE levels and suggest that dietary interventions may have merit as a rational prevention strategy for AD. Moreover, the mechanisms by which high-fat diet and other peripheral signals control central ApoE levels have not yet been elucidated, and future studies will have to address these questions.
